# Quercetin attenuates the progression of monocrotaline-induced pulmonary hypertension in rats

**DOI:** 10.1016/S1674-8301(12)60018-9

**Published:** 2012-03

**Authors:** Hanhua Gao, Can Chen, Shi'an Huang, Bo Li

**Affiliations:** Department of Cardiology, the Affiliated Hospital of Guangdong Medical College, Zhanjiang, Guangdong 524001, China.

**Keywords:** pulmonary arterial hypertension, monocrotaline, quercetin, PCNA

## Abstract

Pulmonary arterial hypertension (PAH) is a progressive disease associated with increased constriction and remodeling of the pulmonary vasculature. Quercetin is a natural flavonoid and has a variety of pharmacological effects including improvement of endothelial cell function. However, its pharmacological effects on pulmonary hypertension have been rarely reported. We sought to observe the protective effect of quercetin in rats with monocrotaline induced PAH. We divided 30 male Sprague-Dawley rats randomly into three groups with ten rats in each group: the monocrotaline group, the quercetin group and the control group. We found that, compared with the controls, the mean pulmonary artery pressure (mPAP) and the right ventricular hypertrophy index in the monocrotaline group were significantly higher (*P* < 0.01). Quercetin caused a significant reduction both in the mPAP and right ventricular hypertrophy index compared with the monocrotaline group (*P* < 0.01) while no difference was found between the quercetin group and the control group (*P* > 0.05). Monocrotaline induced a marked increase in the wall thickness (WT) in small and mid-sized pulmonary arteries compared with the controls (*P* < 0.01). Monocrotaline also induced a marked increase in the wall area (WA) in small [(56.38±6.65)% in monocrotaline *vs*. (19.80±4.63)% in control] and mid-sized [(43.71±5.38)% in monocrotaline *vs*. (14.24±3.66)% in control] pulmonary arteries (*P* < 0.01). Quercetin treatment markedly reduced monocrotaline induced increase in both WT and WA (*P* < 0.01), which, however, still remained significantly elevated compared with those of the controls (*P* < 0.01). Furthermore, compared with controls, proliferating cell nuclear antigen (PCNA) expression in the pulmonary artery tissues was markedly increased by monocrotaline [(45.59±1.27) in monocrotaline *vs*. (9.64±0.69) in controls], which was significantly attenuated by quercetin. Our animal experiment indicated that quercetin could have protective effects on monocrotaline-induced PAH.

## INTRODUCTION

Pulmonary arterial hypertension (PAH) is a progressive disease associated with increased constriction and remodeling of the pulmonary vasculature, ultimately leading to right heart failure with a mean survival time typically less than 3 years. PAH can be diagnosed by measurement of the mean pulmonary artery pressure (mPAP) via right heart catheterization. PAH is diagnosed when the mPAP is over 25 mmHg (resting) or 30 mmHg (motion state) and the pulmonary capillary pressure or left arterial pressure is less than 15 mmHg. Clinical manifestations of pulmonary hypertension are myriad. The current lack of knowledge about PAH and effective diagnostic methods for PAH leads to treatment delays and poor prognosis, and patients finally succumb to right heart failure.

Monocrotaline is a poisonous crystalline alkaloid C_16_H_23_NO_6_ in a leguminous plant of the genus *Crotalaria* (*C. spectabilis*) and in the same genus of other plants. It is metabolized into monocrotaline pyrrole in the rat liver[1]. Monocrotaline pyrrole travels to the lungs through the blood and targets pulmonary artery endothelial cells, resulting in the degeneration, necrosis, and loss of pulmonary artery endothelial cells and thrombosis of the pulmonary artery. Then, the vascular elastic membrane becomes ruptured and vascular smooth muscle cells migrate and proliferate, resulting in pulmonary artery intimal hyperplasia and pulmonary vascular remodeling. Finally, pulmonary arterial pressure was persistently increased[2]. Monocrotaline induced PAH is similar to human pulmonary hypertension.

Quercetin is a natural flavonoid, which is widely distributed in fruits, vegetables and tea, and has a variety of pharmacological effects including inhibition of platelet aggregation[3], improvement of endothelial cell function[4], inhibition of cell proliferation[5] and induction of apoptosis[6], and inhibition of cancer cell growth[7]. Therefore, quercetin has been widely used for anticancer treatment[8]. However, its pharmacological effects on pulmonary hypertension have been rarely reported.

The proliferating cell nuclear antigen (PCNA) is a nuclear protein and a DNA polymerase cofactor. Speir *et al*.[9] found that cell proliferation involves a variety of signaling pathways, which converge at the junction of G_1_/S phase. PCNA expression is necessary for cells to enter the cell cycle to process DNA replication and proliferation. In this study, we sought to investigate whether quercetin could alleviate monocrotaline-induced pulmonary hypertension of rats.

## MATERIALS AND METHODS

### Preparation of animal model and grouping

The experimental protocol was approved by the local institutional review board at the authors' affiliated institution and animal study was carried out in accordance with the established guidelines at the Affiliated Hospital, Guangzhou Medical College, Zhanjiang, Guangdong, China. Thirty clean grade male Sprague-Dawley rats, weighing from 250 to 325 g, were obtained from the Experimental Animal Center of Guangdong Medical College. Rats were randomly divided into three groups: the monocrotaline group, the quercetin group and the control group. Rat pulmonary hypertension was established as described by Cowan *et al*.[10] by a single subcutaneous injection of monocrotaline (50 mg/kg) (Sigma-Aldrich Co., St. Louis, MO, USA) and the control rats were injected with normal saline. In the quercetin group, the rats were given quercetin [100 mg/(kg·d)], (Shanghai Chemical Reagent Co., Shanghai, China) for 21 d on the second day after rats were injected with monocrotaline, while the rats in the other groups were given normal saline instead. After 21 d, the three groups were raised for additional 20 d.

The mPAP was measured at the 41^st^ d after injection of monocrotaline. The rats were anesthetized by pentobarbital and fixed on operating table. The left carotid vein was isolated and after a small incision was made, a coronary guide wire was advanced to the pulmonary artery through the incision under the C-arm X-ray. Then, a manometer catheter was advanced to the pulmonary artery through the wire. The other end of the catheter was connected with the bio-signal acquisition processor without the wire and data on the mPAP was then directly collected[11].

### Right ventricular hypertrophy index

The rats were sacrificed by bloodletting after determination of the mPAP. The left lungs were removed and embedded in paraffin after fixing by 4% paraformaldehyde. The right ventricle (RV) and left ventricle plus septum (LV+S) were weighed using an electronic balance as described by Julian[12] and the right ventricular hypertrophy index was calculated as the weight ratio of RV/(LV+S), and used to describe the degree of right ventricular hypertrophy.

### Wall thickness and wall area

Pulmonary vascular remodeling was evaluated by determining wall thickness (WT) and wall area (WA) according to Barth's method[13]. The left lungs were paraffin-embedded and sectioned at a thickness of 5 µm and stained with hematoxylin-eosin. Ten pulmonary arteries were examined for structural integrity using a morphometric image analysis system independently by two pathologists who were blinded to animal grouping. WT, vessel diameter (ED), the average vessel area (TA) and lumen area (IA) were determined. WT(%) = (2×WT / ED)×100%, and WA(%) = (TA-IA) / TA×100%.

### Immunohistochemistry

Immunohistochemistry was performed using antibodies against PCNA (Shanghai Chemical Reagent Co., Shanghai, China). By using an image analysis system, 10 random samples of each left lung were examined. The number of all blood vessel walls and PCNA stained smooth muscle cell was counted. PCNA proliferation degree (%) = (PCNA positive cells / total cells)×100%[14].

### Statistical analysis

All data in the study were evaluated with the SPSS 10.0 software. Data were expressed as the mean± standard deviation. The comparison between the three groups of data used one-way ANOVA test. Homogeneity of variance was tested by Levene's test. Pairwise comparison between groups used SNK-*q* test. A *P* < 0.05 was considered to be statistically significant.

## RESULTS

### Quercetin antagonizes monocrotaline-induced PAH in rats

We established monocrotaline-induced rat PAH model and measurement of the mPAP showed that compared with the controls, the mPAP in the monocrotaline group was significantly higher (*P* < 0.01), suggesting that the PAH model was successfully established. The right ventricular hypertrophy index was also significantly higher (*P* < 0.01, ***Table 1***). Treatment with quercetin caused a significant reduction both in the mPAP and right ventricular hypertrophy index compared with the monocrotaline group (*P* < 0.01), while no difference was found between the quercetin group and the control group (*P* > 0.05).

**Table 1:** The mean pulmonary artery pressure (mPAP) and right ventricular hypertrophy index

Group	mPAP (mmHg)	Right ventricular hypertrophy index
The control group	14.57 ± 1.59	0.252 ± 0.020
The monocrotaline group	42.13 ± 6.28*	0.529 ± 0.107*
The quercetin group	23.32 ± 3.85**	0.412 ± 0.114**

*Compared with other groups, *P* < 0.01; **compared with the monocrotaline group, *P* < 0.01.

### Quercetin attenuates monocrotaline-induced increase in the wall thickness and area of the heart in rats

**Fig. 1:**
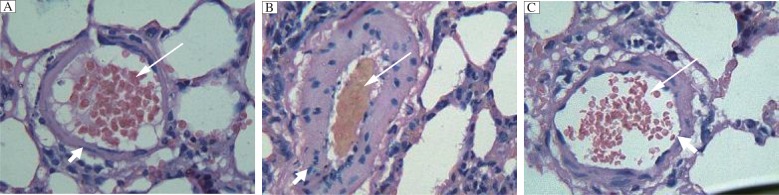
Hematoxylin-eosin staining of the pulmonary artery in the control group (A), rats treated with monocrotaline (B), or with monocrotaline and quercetin (C). The wall in monocrotaline treated rats was thicker than that in controls, which was attenuated by quercetin. Long arrow indicates vessel lumen and short arrow indicates vessel wall. (×400)

We further examined the WT and WA of hearts in these rats. We found that monocrotaline induced a marked increase in the WT in small pulmonary arteries compared with the controls, [(39.66±5.58)% in monocrotaline *vs* (13.40±2.84)% in control (***Table 2***, ***Fig. 1***, *P* < 0.01)]. Similar findings were also observed in mid-sized pulmonary arteries, [(24.04±3.50)% in monocrotaline *vs* (10.59±1.89)% in control (*P* < 0.01)]. Monocrotaline also induced a marked increase in the WA in small [(56.38±6.65)% in monocrotaline *vs*. (19.8±4.63)% in control] and mid-sized [(43.71±5.38)% in monocrotaline *vs*. (14.24±3.66)% in control] pulmonary arteries (*P* < 0.01). Quercetin treatment markedly reduced monocrotaline induced increase in both WT and WA (*P* < 0.01), which, however, still remained significantly elevated compared with those of the controls (*P* < 0.01).

**Table 2:** Wall thickness (WT) and wall area (WA)

Group	Small pulmonary artery		Mid-sized pulmonary artery	
	WT	WA	WT	WA
The control group	13.40 ± 2.84	19.80 ± 4.63	10.59 ± 1.89	14.24 ± 3.66
The monocrotaline group	39.66 ± 5.58*	56.38 ± 6.65*	24.04 ± 3.50*	43.71 ± 5.38*
The quercetin group	23.63 ± 4.31**	38.08 ± 3.98**	16.04 ± 2.50**	27.89 ± 4.30**

*Compared with other groups, *P* < 0.01; **compared with the control group, *P* < 0.01.

(%)

### Quercetin attenuates monocrotaline induced increase in PCNA expression in rat pulmonary artery

**Fig. 2:**
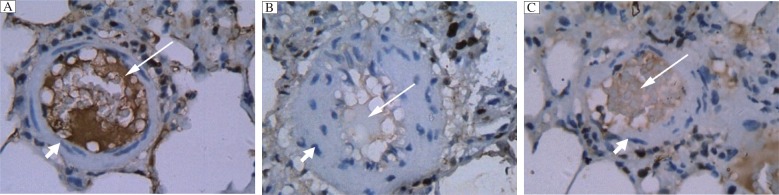
Immunohistochemistry of the pulmonary artery. Tissue sections of the pulmonary artery from rats in the control group (A), the monocrotaline group (B) and the quercetin group (C) were immunohistochemically stained with anti-PCNA antibodies. Long arrow indicates vessel lumen and short arrow indicates vessel wall. (×400)

We examined whether quercetin affected the proliferation of the pulmonary artery tissues treated with monocrotaline by immunohistochemistry using anti-PCNA antibodies. We found that, compared with controls, PCNA expression in the pulmonary artery tissues was markedly increased by monocrotaline, (45.59±1.27) in monocrotaline *vs*. (9.64±0.69) in controls, *P* < 0.01 (***Table 3***, ***Fig. 2***). Quercetin could significantly attenuated monocrotaline induced increase in PCNA expression in the pulmonary artery tissues (*P* < 0.01). However, the expression of PCNA still remained elevated in the quercetin group compared with the control group (*P* < 0.05).

**Table 3:** PCNA expression in rat pulmonary artery

Group	PCNA expression
The control group	9.64 ± 0.69
The monocrotaline group	45.59 ± 1.27*
The quercetin group	17.69 ± 2.85^#,&^

*Compared with other groups, *P* < 0.01; ^#^compared with the monocrotaline group, *P* < 0.01; ^&^compared with the control group, *P* < 0.05.

## DISCUSSION

The clinical manifestations of pulmonary hypertension are breathing difficulty, progressive decline in motor function, and the patient finally dies of right heart failure. Pulmonary vasoconstriction[15], primary thrombus[16] and pulmonary vascular remodeling[17] are the main pathological changes of pulmonary hypertension. Pulmonary hypertension is a complex disease and its pathogenesis has not yet been fully elucidated. The current study shows that pulmonary vasoconstriction is caused by endothelial dysfunction, which is losing the balance between vasodilation (such as nitric oxide and prostacyclin) and vasoconstriction (such as endothelin-1). As a result of pulmonary hypertension, the pulmonary vascular endothelial cells are swelling or even become necrotic. As a consequence, the collagen fibers become exposed, which could cause platelet activation, adhesion and aggregation, and the formation of primary thrombus. Pulmonary vascular remodeling results in thickening of the small artery wall and pulmonary artery stenosis, leading to irreversible pulmonary hypertension. Previous studies have shown that in pulmonary hypertension, pulmonary vascular remodeling is caused by the loss of the balance of cellular proliferation and apoptosis[18]. Pulmonary artery smooth muscle cell proliferation in turn causes wall-thickening and stenosis[19]. The undue proliferation of endothelial cells leads to the thickening of the pulmonary artery intima. It can be inferred that inhibition of the proliferation of pulmonary artery smooth muscle cells and endothelial cells, and promoting apoptosis, may reduce pulmonary artery pressure or reverse pulmonary vascular remodeling, in the hope that PAH can be ultimately cured.

Our results showed that quercetin given for 21 d on the second day after rats were injected with monocrotaline can significantly reduce the mPAP and right ventricular hypertrophy, suggesting that quercetin has a protective effect on pulmonary hypertension. The WT and WA of small pulmonary arteries in the quercetin group were also significantly decreased compared with those in the monocrotaline group, which indicated that quercetin exerted a protective effect on pulmonary hypertension, which may be related to the improvement and reversal of pulmonary vascular remodeling. In this study, another set of data showed that PCNA expression in pulmonary artery smooth muscle cells in the monocrotaline group was significantly higher than that of the control group. It indicated that pulmonary artery smooth muscle cells proliferated significantly after monocrotaline-induced pulmonary hypertension. After quercetin treatment, PCNA expression in pulmonary arterial smooth muscle cells in the quercetin group was significantly lower than that in the monocrotaline group. It indicated that the proliferation of pulmonary artery smooth muscle cells was inhibited. We could infer that quercetin inhibits the proliferation of pulmonary artery smooth muscle cells and could therefore reverse pulmonary vascular remodeling and alleviate pulmonary hypertension, which may lead to improved prognosis of the patient. It may be the reason why quercetin plays a protective role in monocrotaline-induced pulmonary hypertension in rats.

At present, there is still no specific drug treatment for pulmonary hypertension. It is of great clinical significance to protect high-risk groups against the illness. In this regard, our data suggested that quercetin may play a promising role in alleviating pulmonary hypertension and possibly reversing its progression.
